# Nutritional Components and Anti-Alcoholic Liver Disease Activity of Selenium-Enriched *Agaricus subrufescens*

**DOI:** 10.3390/foods15111833

**Published:** 2026-05-22

**Authors:** Hua Chen, Ziyi Wang, Conghui Zhang, Shien Wang, Linghong Zeng, Fang Zheng, Haichen Huang, Jiali Deng, Xucong Lv, Penghu Liu

**Affiliations:** 1 National Engineering Research Center of JUNCAO Technology, College of Life Sciences, Fujian Agriculture and Forestry University, 15 Shangxiadian Road, Fuzhou 320002, China; 2 Institute of Animal Husbandryand Veterinary Medicine, Fujian Academy of Agricultural Sciences, 247 Pu-Dang Road, Fuzhou 350013, China; fjch1976@163.com; 3 Institute of Food Science and Technology, College of Biological Science and Technology, Fuzhou University, Fuzhou 350108, China; 4 International College of JUNCAO Science, Fujian Agriculture and Forestry University, 15 Shangxiadian Road, Fuzhou 350002, China

**Keywords:** *Agaricus subrufescens*, Se, alcoholic liver disease, metabolomics

## Abstract

*Agaricus subrufescens* (AS) is a medicinal mushroom with notable bioactivity and the capacity to accumulate trace elements. In this study, selenium-enriched *A. subrufescens* (SAS) was cultivated, and its protective effects against alcoholic liver disease (ALD) were investigated, with an emphasis on clarifying the underlying mechanisms. The results showed that the yield and antioxidant capacity of mushrooms in a 10 mg·kg^−1^ Se treatment group were increased. Nutritional analysis revealed that SAS contained considerable levels of crude protein (350.00 g·kg^−1^), crude fiber (7.8%), free amino acids (250.20 g·kg^−1^), and other bioactive constituents. Furthermore, the hepatoprotective effects of AS/SAS were studied in male Kunming mice with alcohol-induced liver injury. The body growth, liver index, serum and liver biochemical parameters, histopathological features of liver, hepatic mRNA levels and liver metabolomics were investigated. The results demonstrated that SAS significantly reduced hepatic lipid accumulation, enhanced antioxidant capacity, regulated the mRNA expression of key genes involved in lipid metabolism, oxidative stress, and inflammatory responses, and modulated liver metabolic characteristics. These findings provide theoretical evidence for the potential of SAS as a functional food against alcohol-induced liver injury.

## 1. Introduction

Selenium (Se) is an essential trace element that serves as a crucial cofactor for antioxidant selenoenzymes [1]. Insufficient selenium has been linked to a higher risk of a range of conditions, such as Creutzfeldt–Jakob disease and cardiovascular disease [2]. Edible mushrooms can absorb inorganic Se from cultivation substrates and convert it into organic Se through biological processes, making them an excellent organic Se carrier for producing Se-enriched products [3]. Both *Pleurotus ostreatus* and *Pleurotus djamor* are capable of efficiently transforming selenite (Se(IV)) or selenate (Se(VI)) present in the cultivation substrate into highly bioavailable organic forms [4]. Rathore et al. [5]. reported that *Calocybe indica* also accumulates selenium when grown on Se-enriched wheat straw. A study reported that biofortification of *P. ostreatus* and *P. eryngii* with Se significantly improved their antioxidant activities [6]. *Agaricus subrufescens* (AS), also known as *Agaricus blazei* or *Agaricus brasiliensis*, is highly regarded worldwide for its edible and medicinal properties, particularly in Brazil, the United States, East Asia, and Europe [7]. Based on the excellent biological activity and ability to absorb beneficial elements of *A. subrufescens*, Se-rich cultivation of *A. subrufescens* (SAS) provides a new approach for the development of organic Se products. Therefore, the effects of different exogenous Se concentrations on the growth of AS remain to be elucidated.

Alcoholic liver disease (ALD) is predominantly attributed to chronic or excessive alcohol consumption. It starts with alcoholic hepatitis and fatty liver and develops into liver fibrosis, cirrhosis, and even hepatocellular carcinoma [8]. It should be noted that in addition to direct alcohol-induced toxicity, overnutrition (e.g., excessive caloric or fat intake) frequently coexists with alcohol consumption in clinical practice and can synergistically aggravate hepatic steatosis [9]. Inflammation and oxidative stress triggered by alcohol are key contributors to the development of ALD [10]. The production of reactive oxygen species (ROS) during alcohol consumption can overwhelm the antioxidant system, resulting in the downregulation of antioxidant genes [11,12]. Moreover, ROS can activate immunological cells, leading to a cascade of events and further exacerbating oxidative stress [13]. Given the limitations of current ALD treatments, natural active products represent a promising alternative for alleviating the disease.

AS has been widely used for decades as a medicinal and functional food to support the treatment of various disorders and promote general health [14]. AS has also been widely recognized for its antioxidant, hepatoprotective, and antiviral properties [15]. Given the antioxidant properties of Se and AS, Se enrichment may enhance its hepatoprotective potential. This study therefore aimed to identify the optimal selenium concentration for SAS cultivation and evaluate the protective effects and mechanisms of SAS against alcohol-induced liver injury. These findings establish a theoretical framework for the application of SAS in managing ALD.

## 2. Materials and Methods

### 2.1. Materials

The cultivated strain of SAS (J51) comes from National Engineering Research Center of JUNCAO Technology at Fujian Agriculture and Forestry University. The chemical reagents (AR) were provided by Sinopharm Chemical Co., Ltd. (Shanghai, China). The serum and liver biochemical kits were all purchased from Nanjing Jiancheng Bioengineering Institute (Nanjing, China). The fluorescence quantitative PCR kits were all from EKERE Biotechnology (Changsha, China). Forty SPF-grade male Kunming mice (38 ± 1.8 g) were purchased from Wushi Experimental Animal Trading Co., Ltd. (Fuzhou, China).

### 2.2. Cultivation Experiments of SAS

The basic formula for cultivating SAS is: 35.7% rice straw, 14.29% cow manure, 13.29% wheat bran, 0.02% KH_2_PO_4_, and 31% CaCO_3_. Sodium selenite was used as an exogenous Se additive for Se-rich cultivation, and six different Se concentrations of 0, 5, 10, 20, 40, and 80 mg·kg^−1^ were set, with 0 mg·kg^−1^ as the control group and three replicates per treatment.

### 2.3. Determination of Growth Characteristics of SAS

The first round of uniformly growing fruiting bodies was picked, and the soil in the stem zone was removed. The number of fruiting bodies, individual mushroom weight, and agronomic traits were recorded.

### 2.4. Determination of Antioxidant-Related Indicators in Fruiting Bodies

An amount of 0.1 g of fruiting body was added to 1 mL of buffer solution, homogenized on ice, centrifuged at 8000 r·min^−1^ and 4 °C for 10 min, then the supernatant was collected and kept on ice for testing. Superoxide dismutase (SOD) and peroxidase (POD) were measured using the NBT method and guaiacol method, respectively [16]. Catalase was determined using the hydrogen peroxide method, and glutathione peroxidase (GSH-P_X_), and glutathione (GSH) were determined by spectrophotometry [17,18,19]. The content of malondialdehyde (MDA) was determined using the indene method and thiobarbituric acid method, respectively [20].

### 2.5. Determination of Se Content in Fruiting Bodies

The Se content of SAS was determined by inductively coupled plasma mass spectrometry (ICP-MS, 8800, Agilent Technologies, Santa Clara, CA, USA) after sample digestion according to the GB 5009.268-2025 standard [21] method (pressure tank digestion). The Se enrichment rate was calculated as follows:

$$Se\;enrichment\;rate\;=\;\frac{Se\;content\;of\;fruiting\;body\;\times \;fruiting\;body\;yield}{Total\;Se\;in\;substrate}\;\times \;100\%$$

### 2.6. Determination of Nutritional Components in Fruiting Bodies

The crude fiber content was determined using the van der Waals washing fiber analysis method, the crude protein content was determined using the Kjeldahl nitrogen determination method, the crude fat content was determined using the Soxhlet extraction method, the crude polysaccharide content was determined using the phenol sulfuric acid method, and the free amino acid content was determined using an amino acid analyzer [22].

### 2.7. Animal Experiments

Mice were acclimatized for 10 days under controlled conditions (24 ± 1 °C, 60 ± 5% humidity, normal day/night cycle) with ad libitum access to basal diet and water. Animal experimental procedures for this study were approved by the Ethics Committee of Institute of Food Science and Technology, Fuzhou University, China (approval no.: FZU-IFST-2021010). The mice were then randomly divided into four groups: control group, model group, AS group and SAS group, with 10 mice in each group. Experimental units were randomly assigned to the control and treatment groups using a simple random allocation sequence generated by a random number generator.

Mushrooms were harvested after reaching commercial-grade requirements (Pileus hemispherical to convex, firm, pale yellow to light brown with fibrous scales; stipe robust; partial veil intact, cap tightly enclosing stipe). Freshly harvested mushrooms were first dried in an oven at 40 °C until constant weight, then ground into a fine powder using a grinder. All experiments were performed using mushrooms from the same harvest and the same batch of drying/processing. The amounts required to complete the present experiment were 225 mg for AS and 225 mg for SAS, respectively. In this study, the powder of AS and SAS (14 mg·kg^−1^ body weight each) were resuspended in distilled water and administered orally at 10 a.m. every day as a protective treatment for 6 weeks. The model group, AS group and SAS group were gavaged with a dose of 50% ethanol (7.5 mL·kg^−1^ body weight each) at 2 p.m. every day. During the experiment, all mice were freely given sufficient feed and water and weighed weekly. At the end of the experiment, blood samples and liver tissues were collected and stored at −80 °C for further analysis.

### 2.8. Determination of Body Weight and Liver Index

Body weights were recorded weekly during the experimental period and at sacrifice. The liver index was calculated as follows:

$$liver\;index\;=\;\frac{liver\;weight}{final\;body\;weight}\;\times \;100\%$$

### 2.9. Serum and Liver Biochemical Analysis

Serum levels of total cholesterol (TC), triglycerides (TG), high-density lipoprotein cholesterol (HDL-C), low-density lipoprotein cholesterol (LDL-C), aspartate aminotransferase (AST), and alanine aminotransferase (ALT) were determined using commercial assay kits. Liver levels of TC, TG, catalase (CAT), GSH, GSH-P_X_, SOD, MDA, alcohol dehydrogenase (ADH), and aldehyde dehydrogenase (ALDH) were determined using commercial assay kits.

### 2.10. Histopathological Examination of Liver

Liver tissues were fixed in 4% paraformaldehyde for 24 h, dehydrated in graded ethanol, embedded in paraffin, and cut into 5 μm sections. Sections were stained with hematoxylin and eosin (H&E) and examined under a light microscope (Olympus, Tokyo, Japan). An independent pathologist, blinded to group allocation, performed semi-quantitative histological evaluation based on four criteria: (1) hepatic lobule and hepatocyte cord integrity; (2) hepatocellular cloudy swelling (scored from no swelling with clear cytoplasm to marked swelling with turbid cytoplasm and disorganized lobules); (3) foamy change (scored from no vacuolization to extensive lipid vacuole accumulation); and (4) nuclear morphology (scored from round, central nuclei with distinct nucleoli to indistinct, displaced, or degenerated nuclei).

### 2.11. RT-qPCR Analysis

Total RNA was extracted from liver using Trizol, then reverse-transcribed into cDNA with a commercial kit (including gDNA eraser). RT-qPCR was performed on a StepOne Real-Time PCR system (Applied Biosystems, Foster City, CA, USA) using SYBR^®^ Green Pro Taq HS (Accurate Biotechnology, Changsha, China). Thermal cycling conditions were: initial denaturation at 95 °C for 30 s, followed by 40 cycles of 95 °C for 5 s, 55 °C for 15 s, and 72 °C for 15 s. β-Actin served as the internal control, and relative gene expression was calculated via the 2^−ΔΔCt^ method. Primer sequences are listed in Table S1.

### 2.12. Metabolomics Analysis of Liver

Liver tissue (25 mg) was homogenized in 500 μL of a mixed solvent (acetonitrile:methanol:water = 2:2:1, *v*/*v*/*v*), vortexed for 1 min, and centrifuged at 10,000 rpm for 15 min (4 °C). The resulting supernatant was evaporated to dryness under nitrogen at 37 °C, redissolved in 200 μL of 50% acetonitrile, and centrifuged at 12,000 rpm for 10 min (4 °C). The supernatant was analyzed using a UPLC-QTOF/MS system (Waters Corporation, Milford, CT, USA). Raw data were processed using MPP software (version 15, Agilent Technologies, Santa Clara, CA, USA) for peak detection, alignment, and identification. Multivariate statistical analyses (principal component analysis, PCA; partial least squares-discriminant analysis, PLS-DA; orthogonal partial least squares-discriminant analysis, OPLS-DA) were performed using SIMCA 15.0 software (Umetrics, Malmö, Sweden). Differentially expressed metabolites were identified using the Human Metabolome Database (http://hmdb.ca, accessed on 15 April 2026) with variable importance in projection (VIP) > 1.0 and *p* < 0.05 as criteria. Pathway enrichment analysis was conducted using MetaboAnalyst 5.0 (http://www.metaboanalyst.ca, accessed on 15 April 2026)).

### 2.13. Statistical Analyses

A one-way analysis of variance (ANOVA), followed by the LSD post hoc test, were used to determine statistical significance, and all analyses were performed using the GraphPad Prism 7.0 software. The values were expressed as mean ± SD, statistical significance was denoted as ^##^ *p* < 0.01 and ^#^ *p* < 0.05, versus the model group, and ** *p* < 0.01 and * *p* < 0.05, versus the control group.

## 3. Results

### 3.1. The Impact of Different Se Concentrations on the Agronomic Traits of Fruiting Bodies

**Figure 1:**
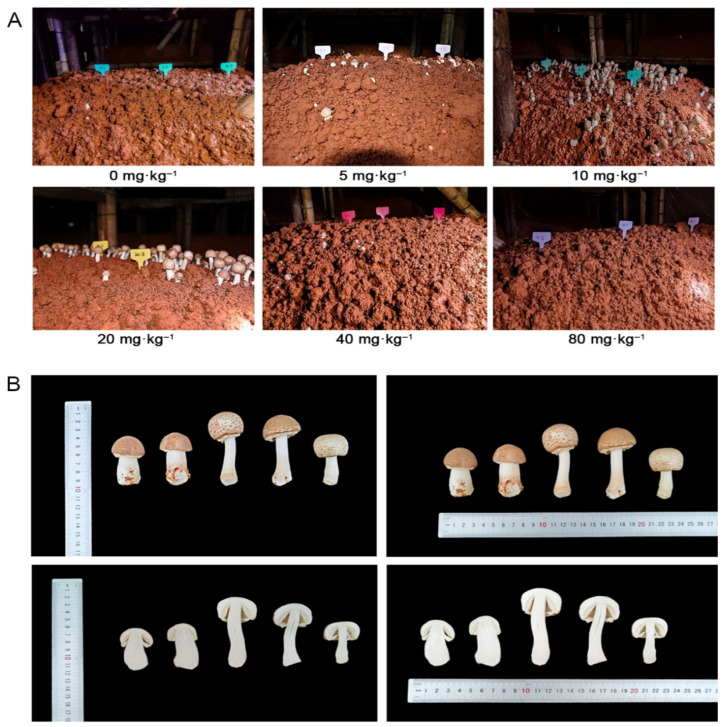
Effects of different concentrations of exogenous selenium on the number of fruiting bodies (**A**). Effects of different concentrations of exogenous selenium on the morphology of fruiting bodies (**B**).

Se supplementation significantly influenced the growth and agronomic characteristics of fruiting bodies. The number of fruiting bodies and yield of AS showed a trend of first increasing and then decreasing with increasing Se concentration, while the weight of individual mushrooms showed the opposite trend (Table S2). The treatment of 10 mg·kg^−1^ and 20 mg·kg^−1^ has proved to increase the number of fruiting bodies and advance the appearance time of fruiting bodies (Figure 1A). As shown in Table S2, the number of mushrooms in the treatment groups of 5, 10 and 20 mg·kg^−1^ was 1.41, 3.65 and 2.86 times that of the control group, and the yields were 1.24, 2.62 and 1.69 times that of the control group, respectively. However, the yield of the 40 mg·kg^−1^ treatment group was 78% of that of the control group (*p* < 0.05), and the fruiting body could not grow in the 80 mg·kg^−1^ treatment group.

The morphology of fruiting bodies was affected by different Se concentrations (Figure 1B). As shown in Table S2, the cap diameter, cap thickness, cap weight, stipe diameter, stipe weight and other traits of fruiting bodies were negatively regulated by Se concentration. It was worth noting that the stipe diameter increased (5–10 mg·kg^−1^) first and then decreased (20–40 mg·kg^−1^) with the increase in Se concentration. In conclusion, the agronomic traits of fruiting bodies were significantly affected by the Se concentration of cultivation material.

### 3.2. The Effects of Different Se Concentrations on Antioxidant System of Fruit Bodies

**Figure 2:**
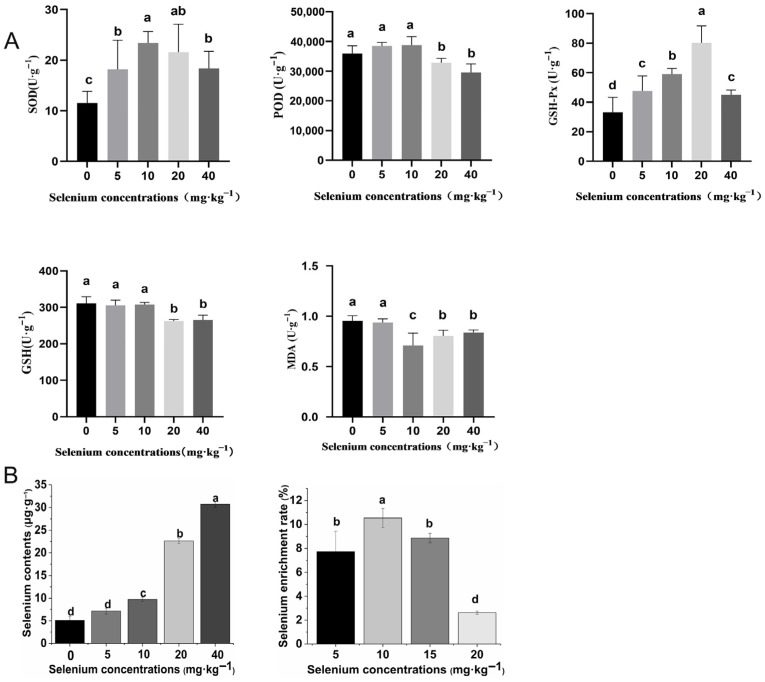
Effects of different concentrations of exogenous selenium on antioxidant systems in the fruiting bodies (**A**). Effects of different exogenous Se additions on selenium content and Se absorption rates in the fruiting bodies (**B**). SOD: superoxide dismutase; POD: peroxidase; GSH-P_X_: glutathione peroxidase; GSH: glutathione; MDA: malondialdehyde. Different letters denote significant differences between groups (*p* < 0.05).

The activities of SOD, POD and GSH-P_X_ increased first and then decreased with the increase in Se concentration. The 10 mg·kg^−1^ treatment resulted in the highest activities of SOD and POD in fruiting bodies (*p* < 0.05), which were 1.02 times and 1.08 times higher than those in the control group, respectively (Figure 2A). The GSH-Px activity of the 20 mg·kg^−1^ treatment was significantly higher than that of the other groups (*p* < 0.05), and was 2.41 times higher than that of the control group.

The content changes in GSH and MDA also reflected the effect of Se on antioxidation systems. As shown in Figure 2A, the content of MDA decreased first (0–10 mg·kg^−1^) and then increased (20–40 mg·kg^−1^) with the concentration of Se. The content of MDA in the 10 mg·kg^−1^ treatment group was significantly lower than that in the other groups (*p* < 0.05). The content of GSH was not significantly affected by low concentration Se treatment, but the GSH content in the 20 mg·kg^−1^ and 40 mg·kg^−1^ treatment was significantly lower than that in the control group (*p* < 0.05). The results showed that Se treatment increased the activity of antioxidant enzymes in fruiting bodies.

### 3.3. The Effects of Different Se Concentrations on Se Contents in Fruiting Bodies

As shown in Figure 2B, the Se content of fruiting bodies is positively correlated with the Se concentration in the cultivation substrate, and the Se content increased from 5.10 μg·g^−1^ to 30.73 μg·g^−1^. However, the Se enrichment rate increased first and then decreased. The 10 mg·kg^−1^ treatment showed that the Se enrichment rate was the highest, which was 10.53% (*p* < 0.05). These results indicated that the effect of exogenous selenium on fruiting body selenium content was not strictly dose-dependent.

### 3.4. The Effects of Different Concentrations of Se on Nutritional Components of Fruiting Bodies

Based on the comprehensive analysis of agronomic traits and physiological biochemical parameters in the fruiting bodies, the nutrients in the fruiting bodies of the 10 mg·kg^−1^ treatment group and the control group were determined (Table S3). The crude polysaccharide content was 0.259%, the crude fiber content was 7.8%, the crude protein content was 350.00 g·kg^−1^, the crude fat content was 15 g·kg^−1^, and the total amino acid (TTA) content was 250.20 g·kg^−1^ in the 10 mg·kg^−1^ treatment group. Compared with the control group, the 10 mg·kg^−1^ treatment significantly reduced the content of crude polysaccharide, crude protein, crude fat and TTA in fruiting bodies, while the content of crude fiber increased by 1.3%. As shown in Table S4, in addition to methionine and tyrosine, the content of other free amino acids decreased in the 10 mg·kg^−1^ treatment. Cystine and arginine decreased significantly, which were 34.62% and 30.00% respectively.

### 3.5. The Effect of SAS on Body Weight and Liver Index in ALD Mice

**Figure 3:**
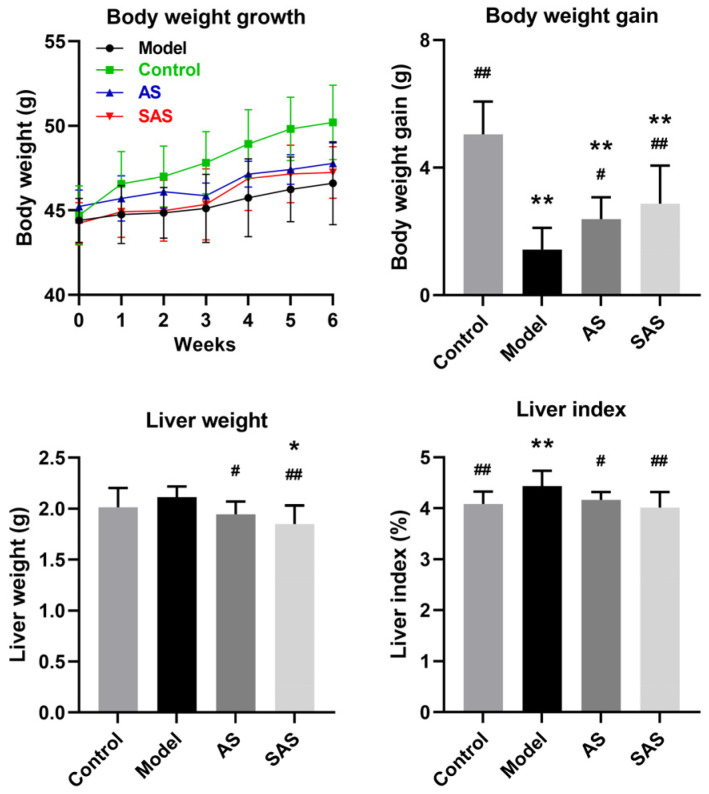
Effects of AS/SAS intervention on the body weight growth and liver index in ALD mice. AS: *Agaricus subrufescens*; SAS: *selenium-enriched A. subrufescens*. Statistical significance was denoted as ^##^ *p* < 0.01 and ^#^ *p* < 0.05 vs. the model group, and ** *p* < 0.01 and * *p* < 0.05 vs. the control group.

As shown in Figure 3, no differences in initial body weight were observed among the experimental groups. After six weeks of alcohol administration, the weight gain of mice in the model group was significantly lighter than that in the control group (*p* < 0.05). However, supplementation with AS/SAS significantly ameliorated alcohol-induced weight loss, with SAS showing a more pronounced effect. The liver index was significantly elevated in the model group (*p* < 0.05), whereas AS/SAS supplementation significantly reduced liver index (*p* < 0.05), with the SAS group showing a lower liver index (4.07%). The above results demonstrate that the effect of SAS was more effective in alleviating the symptoms of mice with excessive alcohol intake.

### 3.6. The Effects of SAS on Histopathological Features of Liver in ALD Mice

**Figure 4:**
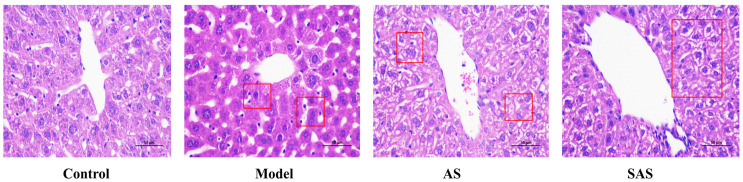
Effects of AS/SAS on liver histological morphology in ALD mice. (×400 magnification; scale bar = 50 μm). AS: *Agaricus subrufescens*; SAS: *selenium-enriched A. subrufescens*.

Histopathological changes in the liver tissues of mice in different experimental groups are shown in Figure 4. Control mice showed well-defined hepatic lobules with orderly hepatocyte cords, round central nuclei, and clear cytoplasm and cell borders. In contrast, excessive alcohol intake led to marked hepatocyte turbidity and nuclear obscurity, indicating intracellular lipid accumulation. As shown in Figure 4, compared with the alcohol model group, the AS intervention group showed markedly reduced liver injury, including alleviated hepatocyte swelling and indistinct nucleoli (left box) and decreased lipid droplets and foam-like changes (right box). However, mild cytoplasmic unevenness and steatosis persisted, and overall recovery was weaker than in the SAS group. Notably, SAS treatment significantly ameliorated alcohol-induced liver injury, with only minimal residual steatosis (right box). The liver morphology was nearly normalized, indicating effective alleviation of hepatic steatosis and hepatocyte damage.

### 3.7. The Effect of SAS on Serum Biochemical Parameters in ALD Mice

**Figure 5:**
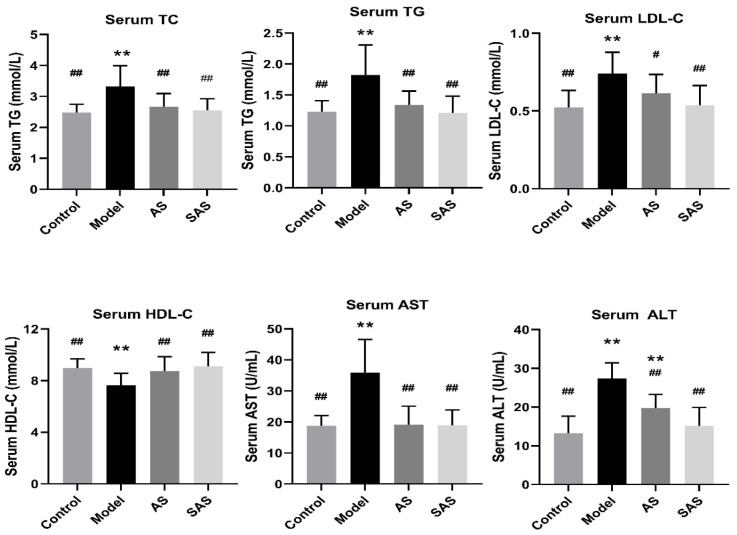
Effects of SAS intervention on the serum biochemical parameters in ALD mice. AS: *Agaricus subrufescens*; SAS: *selenium-enriched A. subrufescens*. TC: total cholesterol; TG: triglycerides; HDL-C: high-density lipoprotein cholesterol; LDL-C: low-density lipoprotein cholesterol (LDL-C), AST: aspartate aminotransferase; ALT: alanine aminotransferase. Statistical significance was denoted as ^##^ *p* < 0.01 and ^#^ *p* < 0.05 vs. the model group, and ** *p* < 0.01 and * *p* < 0.05 vs. the control group.

Serum biochemical indexes can be used as useful and important indexes in the diagnosis of alcoholic liver disease. Alcohol exposure significantly increased serum TC, TG, LDL-C, AST, and ALT levels and decreased HDL-C levels, confirming successful establishment of the ALD model (Figure 5). As shown in Figure 5, after six weeks of AS and SAS intervention, the serum levels of TC, TG, LDL-C were significantly decreased compared with those in the model group, while the serum HDL-C level was significantly increased, especially in the group of SAS. In addition, both AS/SAS supplementation significantly reduced AST and ALT levels (*p* < 0.01), with SAS exhibiting superior efficacy. The therapeutic effect of SAS is stronger than that of AS, which revealed that Se enrichment helps enhance the beneficial effects of AS.

### 3.8. The Effect of SAS on Liver Biochemical Parameters in ALD Mice

**Figure 6:**
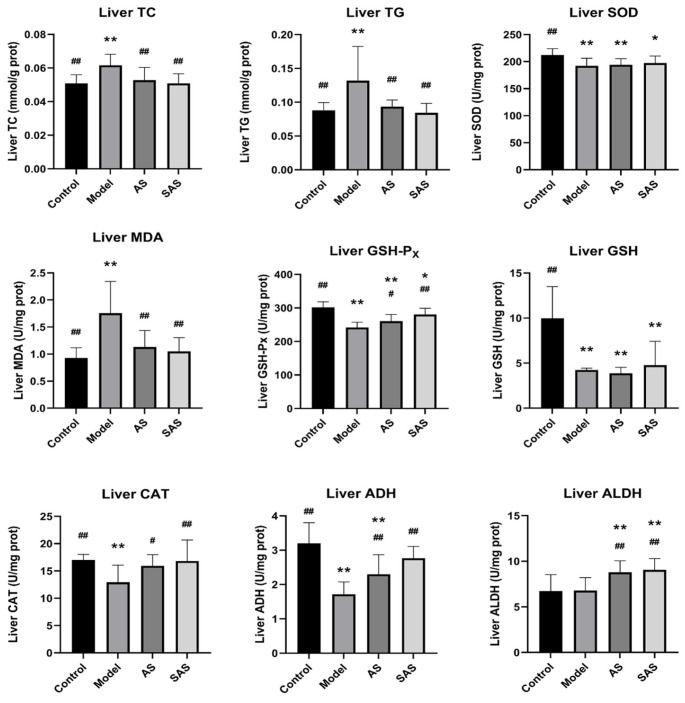
Effects of SAS intervention on the liver biochemical parameters in ALD mice. AS: *Agaricus subrufescens*; SAS: *selenium-enriched A. subrufescens*. TC: total cholesterol; TG: triglycerides; SOD: superoxide dismutase; MDA: malondialdehyde; GSH-P_X_: glutathione peroxidase; GSH: glutathione; CAT: catalase; ADH: alcohol dehydrogenase; ALDH: aldehyde dehydrogenase. Statistical significance was denoted as ^##^ *p* < 0.01 and ^#^ *p* < 0.05 vs. the model group, and ** *p* < 0.01 and * *p* < 0.05 vs. the control group.

As a primary site of alcohol metabolism, the liver may experience abnormally elevated TC and TG levels following chronic excessive alcohol intake, leading to metabolic dysfunction. As shown in Figure 6, the levels of TC and TG in the liver of mice in the model group were significantly higher than those in other groups. After six weeks of AS/SAS administration, the hepatic TC and TG levels significantly reduced in mice exposed to alcohol intake. The oxidative stress status reflects the degree of liver injury induced by excessive alcohol consumption, and those oxidative stress-related parameters including SOD, MDA, GSH-P_X_, CAT, GSH in the liver were also measured. As expected, AS and SAS significantly reversed the decrease in antioxidants (GSH-P_X_, CAT, SOD, GSH) and alcohol-metabolizing enzyme (ADH, ALDH) levels, and the increase in lipid oxidation indicator (MDA). It was worth mentioning that the antioxidant levels of Se-modified AS were significantly higher than those of AS.

### 3.9. The Effect of SAS on Hepatic mRNA Levels in ALD Mice

To explore the mechanism underlying the protective effect of SAS against alcohol-induced liver injury, the mRNA levels of alcohol metabolism-related genes were assessed by RT-qPCR (Figure S1). The genes related to lipid metabolism were investigated in this study including the genes of CD36 molecule (*Cd36*), acyl-coA oxidase 1 (*Acox1*), carnitine palmitoyltransferase1 (*Cpt-1*), peroxisome proliferator-activated receptor α (*Ppar-α*), acyl-coA synthetase long-chain family member 1 (*Acsl1*), catalase (*Cat*) and fatty acid synthase (*Fasn*). In the model group, the mRNA levels of *Acox1*, *Acsl1*, *Cat*, *Ppar-α*, *Cpt-1* were obviously down-regulated, and the mRNA levels of *Cd36*, *Fasn* were obviously up-regulated, which suggested that excessive alcohol can impact lipid metabolism and provoke hepatic cholesterol accumulation. The genes related to oxidative stress were investigated in this study including the genes of nuclear factor erythroid 2 like 2 (*Nrf2*), heme oxygenase-1 (*HO-1*), superoxide dismutase-1 (*Sod1*) and glutathione peroxidase (*GSH-P*_X_). Compared with healthy mice, the transcription levels of genes related to oxidative stress and antioxidant defense in the alcohol group were significantly decreased. The mRNA levels of *Nrf2*, *HO-1*, *Sod1* and *GSH-Px* were found to significantly increase after AS/SAS intervention. ADH2 and ALDH2 are important enzyme activities associated with alcohol metabolism, the levels of *Adh2* and *Aldh2* mRNA were decreased in the alcohol group. Notably, treatment with both AS and SAS effectively reversed the abnormal transcriptional changes observed; however, SAS demonstrated a significantly greater impact compared to AS.

### 3.10. The Effects of SAS on Liver Metabolomic Profiling in ALD Mice

**Figure 7:**
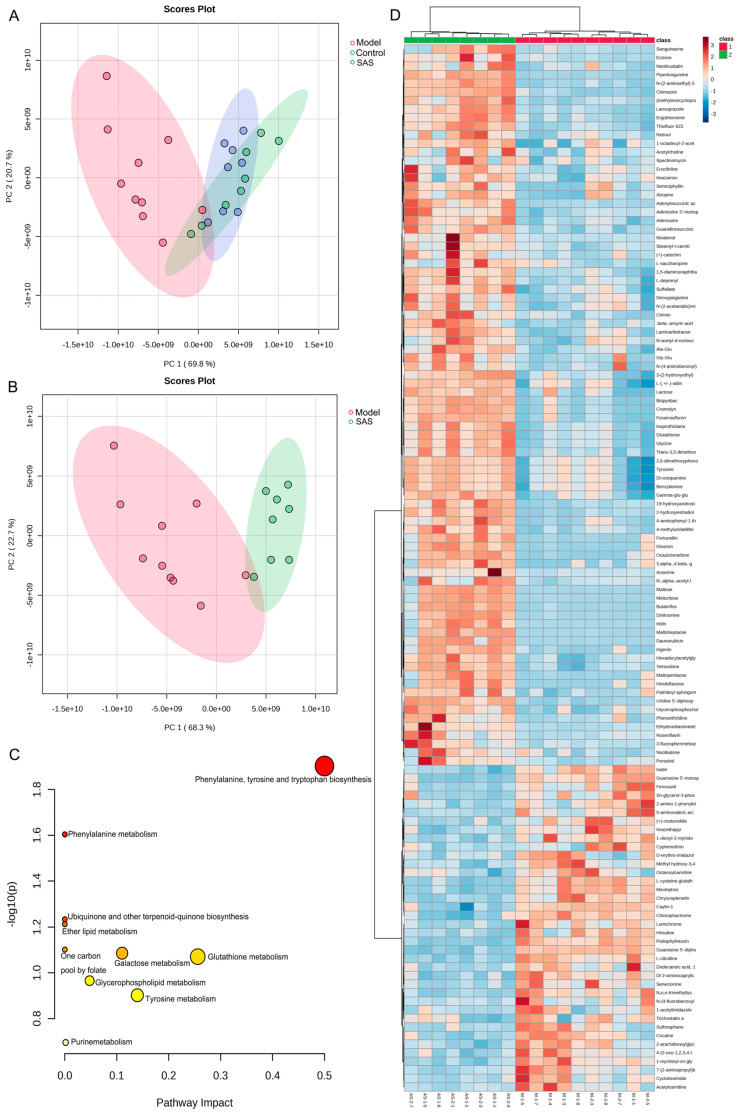
PCA analysis of model group, control group and SAS group (**A**). PCA (principal component analysis) of model group and SAS group (**B**). Pathway analysis of differential liver metabolites from the model and SAS groups (**C**). The differential liver metabolites screened between the model and SAS groups (**D**). SAS: *selenium-enriched A. subrufescens*.

To elucidate endogenous metabolic variations in the liver of ALD mice following 6 weeks of SAS administration, untargeted metabolomics analysis was conducted. In the PCA plot, PC1, PC2, and PC3 accounted for 69.8%, 20.7%, and 5.7%, respectively. The PCA plot displayed an obvious separation clustering between samples from the control and model groups, suggesting the model group mice showed specific metabolic characteristics compared with the model group. Importantly, SAS intervention effectively altered the liver metabolomic profile in ALD mice, which resembled the control group (Figure 7A). Subsequently, the PCA plot displayed an obvious separation clustering between samples from the SAS and model groups (Figure 7B). A total of 122 potential markers were screened and identified in the model and SAS groups (Figure 7D). Among these, the concentrations of sanguinarine, ectoine, neolinustatin, piperlongumine, N-(2-aminoethyl)-5, clomazon, methylenecyclopro, lansoprazole, ergothioneine, thiofluor 623, retinol, l-octadecyl-2-acet, acetylcholine, spectinomycin, erucifoline, lmazamox, seneciphyllin, atropine, adenylosuccinic ac, adenosine 3′-monop, adenosine, guanidinosuccinic, nivalenol, stearoyl-l-carniti, (+)-catechin, l-saccharopine, 1,5-diaminonaphtha, l-deprenyI, sulfallate, and deoxypeganine were significantly increased in the SAS group compared with the model group, but the concentrations of isatin, guanosine 5′-monop, fenoxanil, sn-glycerol-3-phos, 2-amino.1-phenylet, S-aminovaleric aci, (+)-costunolide, imazethapyr, 1-oleoyl-2-myristo, cyphenothrin, D-erythro-imidazol, methyl hydroxy-3,4, octanoylcamitine, L-cysteine-glutath, mevinphos, chrysosplenetin, caylin-1, chlorophacinone, lumichrome, hirsutine podophyllotoxin, guanosine 5′-dipho, L-citrulline, dodecanoic acid, 1, Dl-2-aminocaprylic, senecionine, N,n,n-trimethyllys, N-(4-fluorobenzoy, 1-acetylimidazole, and trichostatin a were significantly reduced. As shown in Figure 7C, SAS intervention regulated the pathways of phenylalanine, tyrosine and tryptophan biosynthesis, tyrosine metabolism, glutathione metabolism, glycerophospholipid metabolism and galactose metabolism in ALD mice.

## 4. Discussion

Global mushroom consumption has increased due to their nutritional benefits. Mushrooms are low in fat yet rich in protein, carbohydrates, dietary fiber, vitamins, and minerals, and they also provide bioactive compounds that boost immunity and support overall health [23]. Mushrooms are known for their ability to accumulate Se, transforming it into various organic species [24]. Among them, the most abundant compound-selenomethionine was identified in *Ganoderma lucidum*, *H. erinaceus*, *L. edodes*, *P. eryngi* and *F. velutipes* [3,25,26,27,28]. Owing to their unique nutritional attributes and capacity to accumulate and metabolize selenium, mushrooms represent a promising dietary source of organic Se. The results showed that the number of fruiting bodies and yield of AS increased first and then decreased with the increase in Se concentration. Fungal growth is stimulated by low selenium concentrations, as found in prior research, but DNA damage and growth inhibition occur when selenium levels are excessive [5,29]. The fruiting bodies of the 10 and 20 mg·kg^−1^ treatment groups demonstrated a thinner and elongated phenotype, while the 40 mg·kg^−1^ group displayed reduced thickness, shorter length, and decreased single mushroom weight. Marliane et al. [30]. revealed that Se concentrations above a certain limit induced stipe elongation and cap size diminishment in fungal fruiting bodies. This phenomenon may be attributed to exogenous Se that promotes primordium formation, while the simultaneous germination of numerous fruiting bodies could lead to nutrient competition, resulting in reduced individual mushroom weight.

Low concentrations of Se exhibit a positive effect on antioxidant capacity [31]. In the study of Se enriched *Ganoderma lucidum* cultivation, it was found that the genes related to antioxidant activity can be regulated by Se to promote growth [32]. This study also showed that the changes in antioxidant capacity of different Se concentrations were consistent with the changes in yield, and there was a positive correlation between them. As the key protective enzyme of the antioxidant system, SOD can reduce reactive oxygen species to H_2_O_2_ and O_2_, and then POD can decompose H_2_O_2_ into H_2_O and O_2_ [33]. Previous research reported that Se acts as an antioxidant at low concentrations and as an oxidant at high concentrations, which has adverse effects on the growth of organisms [34]. The results of this study showed that the high concentration of Se treatment led to the increase in MDA content, indicating that the high concentration of Se treatment caused stress on the fruiting body, produced oxidative stress, slowed down or even inhibited the growth of fruiting body. Se toxicity might be due to the substitution of Se for sulfur in proteins or may be due to inhibition of methylation [31]. However, the results demonstrated that Se treatment elicited a dichotomous modulation of antioxidant enzymes in the fruiting bodies. This may be that the optimal Se concentration of some oxidases was lower than 10 mg·kg^−1^.

The Se content of fruiting body is positively correlated with the concentration of exogenous Se, which was consistent with the results of Song et al. [35] on Se enriched cultivation of *Phellinus igniarius* hyphae. However, the Se enrichment factor increased first and then decreased. The 10 mg·kg^−1^ treatment showed that the Se enrichment factor was the highest. The initial increase in Se enrichment factor (up to 10 mg·kg^−1^) indicates efficient Se uptake and biotransformation within the mushroom’s tolerance capacity. The subsequent decline at higher Se levels (20–40 mg·kg^−1^) suggests saturation of detoxification pathways and the onset of Se toxicity, as evidenced by reduced yield and antioxidant enzyme activities. Therefore, 10 mg·kg^−1^ represents the optimal balance between Se enrichment and mushroom health under our cultivation conditions. It should be noted that all other cultivation parameters (substrate composition, moisture, temperature, light cycle, etc.) were kept constant across the different Se concentration groups, ensuring that the observed effects are solely attributable to the Se dose. Treatment with suitable selenium concentrations yields a range of beneficial effects in mushrooms [36]. Selenium treatment can alter the metabolism of carbohydrates, proteins, and lipids, along with other physiological processes, thereby affecting mushroom biomass yield and nutrient composition [37]. This study observed that Se treatment reduced crude polysaccharide, crude protein, crude fat and a variety of free amino acids. A previous study has shown that selenium influences a wide range of fungal metabolic pathways, such as glycolysis, binding of ATP, metals, nucleosides, and nucleotides, along with protein conformation, stress responses, and signaling cascades [38].

ALD reportedly affects 25% of the population, making it the second leading cause of liver disease after viral hepatitis [39]. Inflammation is a key factor in the onset and progression of alcoholic liver disease [40]. Mushrooms have long been valued in traditional medicine for their numerous health benefits [41]. AS has been reported to produce a variety of bioactive compounds, such as polysaccharides, polyphenols and agarol [42]. This species exerts preventive effects against various diseases, including cancer, chronic hepatitis, diabetes, atherosclerosis, and hypercholesterolemia [43].

Excessive ethanol intake disrupts the tricarboxylic acid cycle and fatty acid oxidation, thereby altering lipid metabolism and resulting in hepatic TG accumulation and hypertriglyceridemia [44]. In this study, the intake of AS/SAS reduced the levels of TG and TC in blood and liver, suggesting that AS/SAS attenuated hepatocyte steatosis. The protective effect was further evidenced by histological analysis of ALD mouse livers, which showed decreased microvesicular steatosis and ballooning after AS/SAS treatment. Previous studies have demonstrated that serum LDL-C and HDL-C levels are strongly linked to the pathogenesis of glucose and lipid metabolism disorders [45]. The results also prove that SAS has a better effect on regulating lipid metabolism. Elevated serum ALT and AST levels, which are key indicators of liver injury, result from increased hepatocyte membrane permeability caused by chronic alcohol consumption [46]. This study observed that the intake of AS/SAS significantly reduced the levels of ALT and AST in the serum of patients with ALD, indicating that AS/SAS restored the function of hepatocytes and alleviated the liver injury caused by ethanol. These results collectively demonstrate that AS/SAS possesses hepatoprotective properties against ethanol-induced liver injury, and the protective effect of SAS was stronger.

Oxidative stress is regarded as a major driver of ALD [47]. Overabundant ROS production can impair or deplete intrinsic antioxidant mechanisms, resulting in a cascade of oxidative stress, hepatocyte apoptosis, lipid peroxidation, and consequent hepatic damage [48]. As an end product of lipid peroxidation, MDA levels indicate the severity of cellular damage. Meanwhile, the key antioxidants SOD, GSH, and CAT function to eliminate free radicals and attenuate oxidative injury [49]. This study observed that the intake of AS/SAS significantly increased the contents of SOD, GSH and Cat in mice, enhanced the antioxidant capacity and counteracted the alcohol-induced liver injury. After treatment with AS/SAS, the liver MDA level was significantly lower than that in the model group, suggesting that AS/SAS could significantly reduce alcohol-induced oxidative damage. The Se is an essential component of the active center of GSH-P_X_ [50]. The activity of GSH-P_X_ was significantly increased after SAS intervention. Low ADH/ALDH activity causes acetaldehyde accumulation, leading to oxidative stress and hepatocyte apoptosis [51]. The intervention of AS/SAS has been shown to enhance the activity of ADH and ALDH, which can reduce the risk of alcohol liver injury. These findings demonstrate that AS/SAS intervention elevates antioxidant enzyme levels, thereby attenuating alcohol-induced oxidative stress in the liver. Notably, SAS exhibited a stronger protective effect.

To elaborate on the potential mechanism by which SAS intervention protects against alcohol-induced liver injury, the transcription levels of genes related to alcohol metabolism were further analyzed by RT-qPCR. *Acsl1* stimulates the transfer of fatty acids and the synthesis of triglycerides, while *Cpt-1* modifies long-chain fatty acyl-CoA into acylcarnitine, participating in the metabolism of fatty acids; it has been widely recognized that *Acox1*, *Cpt-1* and *Ppar-α* are regulators controlling the *β*-oxidation of fatty acids. *Ppar-α*, in particular, can regulate the liver lipid homeostasis by modulating the expression of *Acox1* [52]. *Cd36* serves as both a lipid transporter and a pattern-recognition receptor on the cell surface. It critically regulates fatty acid homeostasis, thereby influencing the progression of hepatic steatosis [53]. *Nrf2* has been extensively investigated as a therapeutic target for ALD due to its role as a master regulator of cellular adaptive antioxidant responses and its ability to facilitate toxin detoxification. Activation of *Nrf2* induces *HO-1* expression, thereby boosting antioxidant defense [54]. ADH2 and ALDH2 are recognized as key rate-limiting enzymes for hepatic alcohol metabolism, and dysregulated expression of their genes is strongly associated with the development of various liver diseases [55]. Interestingly, our results found that AS/SAS intervention remarkably restrained the mRNA transcription of *Fasn*, and *Cd36*, but increased the mRNA transcription of *Acox1*, *Acsl1*, *Cpt-1*, *Adh2*, *Aldh2*, *Nrf-2*, *GSH-Px*, *HO-1*, *Ppar-α*, *Sod1* and *Cat*. These results indicate that AS and SAS activate the Nrf2-mediated antioxidant pathway, as evidenced by upregulation of *Nrf2*, *HO-1*, *GSH-Px*, *Sod1*, and *Cat*. Nrf2 activation is known to protect against oxidative stress-induced mitochondrial dysfunction, which is a key event in alcohol-related liver injury [56,57]. Although we did not directly measure mitochondrial parameters (e.g., membrane potential, ATP, or mitochondrial ROS), the enhanced antioxidant response strongly suggests preserved mitochondrial integrity. The superior effect of SAS may be attributed to selenium-dependent upregulation of selenoproteins such as GSH-Px, further enhancing mitochondrial protection.

In this study, SAS intervention significantly up-regulated the sanguinarine, piperlongumine, (+)-catechin, and glutathione in ALD mice. Sanguinarine is a phenylalanine-derived alkaloid, and its increased content may be related to the upregulation of the phenylalanine metabolic pathway by SAS. Previous findings demonstrate that supplementation of sanguinarine in diets ameliorates hepatic lipid accumulation, bile acid dysregulation, and tissue damage, ultimately enhancing liver and intestinal health [58]. Piperlongumine effectively combats metabolic dysfunction-associated fatty liver disease induced by a high-fat diet and improves metabolic characteristics in mice [59]. The enhanced protection provided by the quercetin–catechin combination against EtOH-induced oxidative stress alleviates acute alcoholic liver disease in rats [60]. The increase in glutathione content was directly related to the activation of the glutathione metabolic pathway. Glutathione metabolism is known to be closely related to oxidative stress and is one of the main metabolic pathways involved in alleviating alcohol injury [61]. In the study, it was found that oral administration of SAS increased the level of glutathione. Therefore, the enhancement of sanguinarine, piperlongumine, (+)-catechin, and glutathione is beneficial for improving liver function. In addition, SAS intervention significantly reduced imazethapyr, podophyllotoxin, and senecionine. Imazethapyr is a toxic substance that can increase hepatic MDA levels, the intervention of SAS can reduce the levels of these toxic substances and subsequently ameliorate liver injury [62]. Previous research had shown that podophyllotoxin activates the cGMP-PKG pathway, inhibiting autophagy and further accelerating pyroptosis, ultimately leading to hepatotoxicity [63]. Senecionine, a toxic pyrrolizidine alkaloid, can result in mitochondrial damage and cell apoptosis in liver cells [64]. These results collectively demonstrate that SAS could improve ethanol-induced liver injury by regulating metabolites.

AS has been reported to produce a variety of bioactive compounds, such as polysaccharides, polyphenols and agarol [42]. Numerous natural plant-derived polysaccharides possess potent anti-ALD activity. For example, *Ganoderma lucidum* polysaccharides alleviate acetaminophen-induced acute liver injury by suppressing oxidative stress and Nrf2-mediated apoptosis [65]. Se-enriched polysaccharide possesses Seleno oxygen with a unique structure and shows higher functional activities than polysaccharide [66]. Se-enriched proteins possess higher bioactivity than native proteins, attributable to the role of selenium as the catalytic core of various selenoenzymes [67]. SAS is rich in Se-enriched polysaccharide and Se-enriched proteins, thus exhibiting superior biological activity compared to the original AS. In the present study, Se-enriched polysaccharides and Se-enriched proteins are considered the pivotal bioactive constituents responsible for the observed hepatoprotective effects. The biosynthesis and accumulation of these key functional components are specifically regulated by selenium enrichment cultivation. Selenium supplementation during culturing directly modulates their content and structural properties, which explains the stronger biological activity of SAS compared with conventional AS well.

## 5. Conclusions

In this study, SAS was successfully cultivated, and 10 mg·kg^−1^ sodium selenite was identified as the optimal concentration for improving fruiting body yield, antioxidant capacity, and Se enrichment efficiency. SAS supplementation suggested a protective effect against alcohol-induced hepatic steatosis, oxidative stress, and inflammation in mice. Mechanistically, SAS restored lipid metabolism, activated Nrf2-mediated antioxidant signaling, enhanced alcohol catabolism, and normalized hepatic metabolic pathways. These findings suggest that SAS may have potential as a natural protective agent against ALD, offering insights for high-value utilization of this agricultural product and a possible basis for its development in functional foods. A limitation of this study is that the mechanism by which selenium enrichment enhances hepatoprotective activity remains unclear, as our data cannot distinguish direct effects of selenium from selenium-induced metabolic changes in the mushroom. Further investigation with appropriate controls is needed.

## Data Availability

The original contributions presented in this study are included in the article/Supplementary Materials. Further inquiries can be directed to the corresponding authors.

## Supplementary Materials

The following supporting information can be downloaded at: https://www.mdpi.com/article/10.3390/foods15111833/s1, Table S1. Primer sequences for RT-qPCR. Table S2. Effects of different concentrations of Se stress on agronomic characters of *A. subrufescens.* Table S3. Effects of exogenous Se on nutrients in *A. subrufescens.* Table S4. Effects of exogenous Se on free amino acids in *A. subrufescens.* Figure S1. Effects of SAS administration on the mRNA levels in livers of ALD mice.
