# Supplementary material for: Nutritional Components and Anti-Alcoholic Liver Disease Activity of Selenium-Enriched Agaricus subrufescens
Source: Foods. 2026 May 22;15(11):1833. doi: 10.3390/foods15111833 (PMC13256301; doi:10.3390/foods15111833)
Supplement: Supplementary file 1 [file foods-15-01833-s001.zip › foods-4305253-supplementary.pdf]

Supplementary data

S1. Methods

Table S1 Primer sequences for RT-qPCR

gene	Forward primer (5′–3′)	Reverse primer (5′–3′)
Acox1	GCCTGCTGTGTGGGTATGTCATT	GTCATGGGCGGGTGCAT
Acsl1	CACTTCTTGCCTCGTTCCAC	GTCGTCCCGCTCTATGACAC
Adh2	AACGGTGAGAAGTTCCCAAAA	ACGACCCCCAGCCTAATACA
Aldh2	ATCCTCGGCTACATCAAATCG	GTCTTTTACGTCCCCGAACAC
Cat	TCA CCC ACG ATA TCA CCA GA	AGC TGA GCC TGA CTC TCC AG
Cd36	ATGGGCTGTGATCGGAACTG	GTCTTCCCAATAAGCATGTCTCC
Cpt-1	TCCATGCATACCAAAGTGGA	TGGTAGGAGAGCAGCACCTT
Fasn	CTGCCACAACTCTGAGGACA	TTCGTACCTCCTTGGCAAAC
GSH-Px	CCACCGTGTATGCCTTCTCC	AGAGAGACGCGACATTCTCAAT
HO-1	AACAAGCAGAACCCAGTCTATGC	AGGTAGCGGGTATATGCGTGGGCC
Nrf2	CCGGGGAACAGAACAGGAAA	ACGTTGTCCCCATTTTTGCG
Ppar-α	TGCCTTCCCTGTGAACTGAC	TGGGGAGAGAGGACAGATGG
Sod1	TTG GCC GTA CAA TGG TGG	CGC AAT CCC AAT CAC TCC AC
β-Actin	TGTTACCAACTGGGACGACA	GGGGTGTTGAAGGTCTCAAA

S2. Results

Table S2 Effects of different concentrations of Se stress on agronomic characters of *A. subrufescens*

Se concentration ($mg.kg^{-1}$)	Single weight (g)	Number of fruiting bodies	Yield (kg)	Cap diameter (cm)	Stipe diameter (cm)	Cap thickness (cm)	Stipe length (cm)	Cap weight (g)	Stipe weight (g)
0	21.59±3.30^{a}	17.00±1.73^{d}	1.00±0.10^{d}	3.53±0.32^{a}	1.69±0.26^{a}	0.71±0.08^{a}	6.73±0.45^{b}	10.72±2.02^{a}	10.81±1.37^{a}
5	19.58±3.30^{a}	24.00±1.00^{c}	1.24±0.05^{c}	3.24±0.32^{b}	1.31±0.23^{b}	0.73±0.12^{a}	6.72±0.62^{b}	10.45±2.01^{a}	9.17±1.62^{b}
10	13.99±3.58^{b}	62.33±1.15^{a}	2.62±0.05^{a}	2.81±0.35^{c}	1.17±0.14^{c}	0.63±0.07^{b}	8.08±0.52^{a}	7.94±1.94^{b}	6.46±1.11^{c}
20	11.61±1.16^{c}	48.67±0.58^{b}	1.69±0.02^{b}	2.78±0.15^{c}	1.10±0.13^{c}	0.59±0.05^{b}	7.68±0.63^{a}	6.56±0.93^{b}	5.12±0.41^{d}
40	15.57±2.21^{b}	16.67±2.31^{d}	0.78±0.11^{e}	3.50±0.28^{a}	1.38±0.15^{b}	0.50±0.07^{c}	5.80±0.52^{c}	10.09±1.37^{a}	5.44±0.99^{d}

Note: different small letters in the same column indicate significant differences ($p<0.05$)

Table S3 Effects of exogenous Se on nutrients in *A. subrufescens*

Nutrients	Control group	10 $mg \cdot kg^{-1}$ treatment group	Decrease (%)
Crude polysaccharide (%)	0.87*	0.26	70.37
Crude fiber (%)	7.70	7.80	1.30
Crude protein ($mg \cdot kg^{-1}$)	383.00*	350.00	8.62
Crude fat ($mg \cdot kg^{-1)}$	20.00*	15.00	25.00
TAA ($mg \cdot kg^{-1}$)	276.40	250.20	9.48

*There was a significant difference between the two groups (p<0.05)

Table S4 Effects of exogenous Se on free amino acids in *A. subrufescens*

Free amino acids	Control group	10 mg·kg^{-1} treatment group	Decrease (%)
Asp	24.50*	21.30	13.06
Thr	12.30	11.40	7.32
Ser	12.50*	11.30	9.60
Glu	46.60*	42.10	9.66
Gly	14.90*	13.10	12.08
Ala	19.50	17.80	8.72
Cys	2.60*	1.70	34.62
Val	14.20	13.00	8.45
Met	21.80	22.70	-4.13
Ile	12.00	11.30	5.83
Leu	19.20	18.10	5.73
Tyr	9.00	9.50	5.56
Phe	11.20	10.50	6.25
Lys	17.60*	15.60	11.36
His	6.10	5.50	9.84
Arg	21.00*	14.70	30.00
Pro	11.40	10.60	7.02

*There was a significant difference between the two groups (p<0.05)

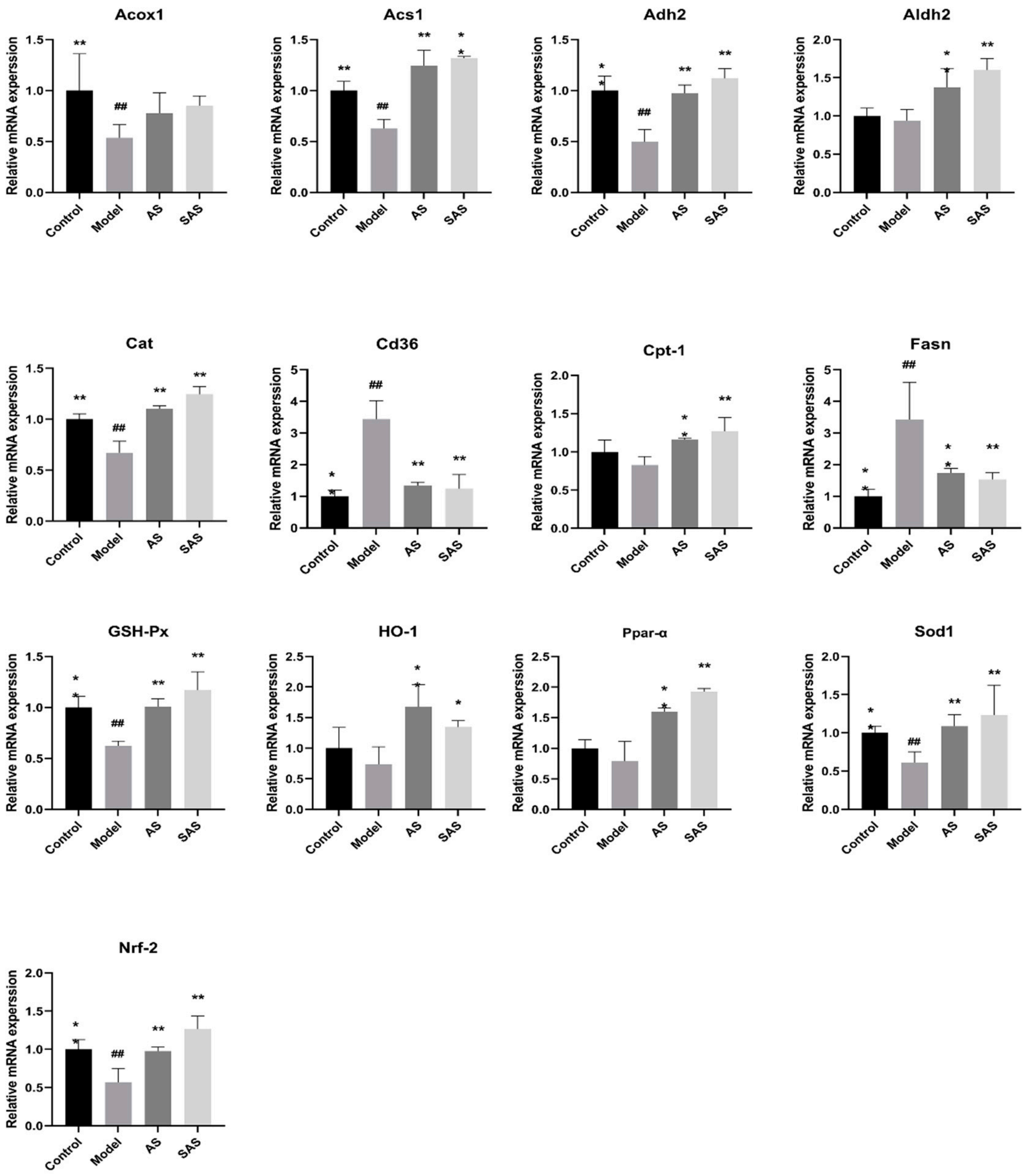

Figure S1. Effects of SAS administration on the mRNA levels in livers of ALD mice. AS: *Agaricus subrufescens*; SAS: *selenium-enriched A. subrufescens*. *Acox1:* acyl-coA oxidase 1; *Acsl1:* acyl-coA synthetase long-chain family member 1; *Adh2*: alcohol dehydrogenase 2; *Aldh2*: aldehyde dehydrogenase 2; *Cat*: catalase; *Cd36*: CD36 molecule; *Cpt-1:* carnitine palmitoyltransferase1; *Fasn:* fatty acid synthase; *GSH-P*x: glutathione peroxidase; *HO-1:* heme oxygenase-1; *Ppar-α:* peroxisome proliferator - activated receptor *α*; *Sod1:* superoxide dismutase-1; *Nrf2:* nuclear factor erythroid 2 like 2. Statistical significance was denoted as ## *p*

< 0.01 and $^{\#}$ $p < 0.05$ vs. the model group; ** $p < 0.01$ and * $p < 0.05$ vs. the control group.
